# Uncovering heterogeneity in mental health changes among first-year medical students

**DOI:** 10.1080/10872981.2024.2317493

**Published:** 2024-02-23

**Authors:** Sabine Polujanski, Ulrike Nett, Thomas Rotthoff, Melissa Oezsoy, Ann-Kathrin Schindler

**Affiliations:** a Medical Didactics and Education Research, DEMEDA (Department of Medical Education), Medical Faculty, University of Augsburg, Augsburg, Germany; b Department of Empirical Educational Research, Faculty of Philosophy and Social Sciences, University of Augsburg, Augsburg, Germany; c Department of Education and Educational Psychology, Faculty of Psychology and Education, Ludwig-Maximilians-University Munich, Munich, Germany

**Keywords:** Medical students, dual-factor model, freshmen, latent profile analysis, mental health changes

## Abstract

**Introduction:**

The initial year of medical school is linked to a decline in mental health. To assess mental health comprehensively, the dual-factor model posits the consideration of both psychopathology (e.g., depression) and positive mental health (e.g., well-being). Previous mental health research among medical students has primarily examined these two factors independently. This study uses the dual-factor approach for a deeper understanding of mental health changes during the first year of medical school.

**Methods:**

Students from eight German medical schools (*N* = 450) were surveyed three times (T0 = entering medical school, T1 = end of the first semester, T2 = end of the second semester) regarding depression (PHQ-9), well-being (subscale of FAHW-12), and general life satisfaction (German Single-Item Scale L1). Latent profile analysis was used to identify distinct mental health groups based on their combinations of psychopathology and positive mental health. We then analysed trajectories descriptively by examining the longitudinal stability and dynamics of mental health group membership during the first year of medical school.

**Results:**

We identified five mental health groups: (1) complete mental health, (2) moderately mentally healthy, (3) symptomatic but content, (4) vulnerable, and (5) troubled. The examination of change trajectories unveiled diverse paths pointing towards both recovery and deterioration. In comparison to the other groups, students belonging to the complete mental health group exhibited greater stability and a higher potential to recover after initial deteriorations in the first semester.

**Conclusions:**

Our study uncovers distinct mental health trajectories in the first year of medical school, emphasizing the crucial role of initial mental health status. Our findings stress the diverse nature of mental health changes in medical students, underscoring the need for tailored prevention strategies. The implications for research and practice are discussed.

## Introduction

Empirical research confirms that medical school is highly challenging. The first year of medical school is already associated with an overall increase in psychopathology (e.g., depression [1–3], burnout [3]), and an overall decrease in positive mental health (PMH) (e.g., emotional health [4]). Previous research has primarily examined these two factors of mental health (psychopathology and PMH) independently. However, from a dual-factor mental health perspective, the exclusive consideration of psychopathology or PMH is insufficient to provide an accurate picture of mental health [5,6]. In contrast to the traditional assumption that the absence of psychopathology means the presence of mental health (or vice versa), the dual-factor model of mental health assumes that psychopathology and PMH are interrelated constructs that lie on distinct continua [5,7]. This implies that an individual can experience high levels of distress and psychological well-being simultaneously. Studies support the necessity to consider both factors when assessing, preventing and intervening in mental health [5–7].

Mental health deteriorations can have negative effects on learning [8], academic performance [9,10] and social functioning [11]. This is particularly worrying given that only a small percentage of affected medical students seek psychiatric support [12,13].

Deteriorations in mental health in the initial year of medical school might be related to emotional and social challenges commonly associated with entering university (e.g., moving to a new city, unfamiliar university learning practices, dealing with high workload, habituation to examination and fostering new relationships [14,15]). Moreover, students enter university with varying levels of mental health in terms of depression [1,2,16], and anxiety [16,17], which might affect these adaptation processes. Furthermore, the rigorous admission criteria for medical school engender a group of high-performing students, potentially fostering a highly competitive atmosphere. This might also contribute to mental morbidity [15].

Besides the focus on either psychopathology *or* PMH, previous research among medical students has paid little attention to the *heterogeneity* in mental health changes. To the best of our knowledge, only two exceptional studies have examined distinct depression development patterns in medical students, highlighting the protective role of individual characteristics [1] and career choice motives [8]. These initial findings emphasize the importance of considering the variability in how depression evolves. This, in turn, aids in gaining a deeper understanding of how depression may originate during medical school or potentially become more pronounced as individuals enter medical school, with the possibility of exacerbation during their later careers.

With the present study, we aim to address those research gaps by investigating medical students’ initial dual-factor mental health status and its changes during the first year of medical school.

### Dual-factor mental health

Contemporary approaches assume, that the lack of psychopathology (symptoms and deficits in functioning, e.g., depression) does not necessarily indicate the presence of PMH, which refers to elevated levels of emotional, social, and psychological well-being [6]. Models that incorporate both factors (psychopathology and PMH) – known as dual-factor (e.g., [5]) or two-continua (e.g., [18]) models of mental health – conceptualize psychopathology and PMH as indicators of mental health that lie on separate but related continua [5,7]. This means that PMH and psychopathology can occur simultaneously.

Based on the dual-factor model, four discrete mental health groups can be distinguished: (1) complete mental health (characterized by high PMH and low psychopathology), (2) symptomatic but content (characterized by high PMH and high psychopathology), (3) vulnerable (characterized by low PMH and low psychopathology) and (4) troubled (characterized by low PMH and high psychopathology [7]). These groups were repeatedly found in different populations, e.g., early elementary students [19]; middle school students [20–22]; high school students [23,24]; college students [25,26] and migrant workers [27].

Numerous studies support the advantage of the dual-factor model, as shown by a scoping review of clinical and non-clinical studies across diverse populations, which found stronger explanatory power for the dual-factor model over unidimensional traditional approaches [28]. Traditional assessment methods have the potential to either overestimate or underestimate individuals’ functioning [7]. For instance, research in schools has unanimously shown that students with *complete mental health* fare better in various academic outcomes compared to those in the other groups [7,21,23]. The v*ulnerable* group – an overlooked group in traditional approaches – is likelier to face academic and behavioral challenges compared to the *complete mental health* group [22]. These findings support the assumption that merely lacking psychopathology is inadequate for optimal functioning [7,21,23]. In addition, among individuals with psychopathology, those with higher PMH (*symptomatic but content*) experienced more favorable outcomes compared to their counterparts with comparable levels of psychopathology but low PMH (*troubled*) [7,23].

Also, studies among college students found support for the dual-factor model revealing variations in (1) hope, gratitude, attention problems, and locus of control [25,29], (2) grit, savoring of positive emotions and self-focused positive rumination [29], (3) academic achievement, interpersonal connectedness, and physical health [30], as well as (4) academic emotions [31] between the sub-groups.

### Longitudinal stability of dual-factor mental health groups

Existing longitudinal studies on dual-factor mental health primarily deal with the question of whether specific dual-factor mental health groups exhibit greater stability or transience than others (e.g., [32]). Comparisons of these studies are challenging due to different grouping methods, differences in the investigated samples, the use of mental health indicators and distinct time intervals. However, all studies – primarily conducted in the school context – consistently demonstrated the highest stability for the *complete mental health* group (64%–86%) regardless of the examined period ranging from five months to three years [19–21,32–34]. Furthermore, all groups showed patterns of instability, with the *vulnerable* [19,20,34], *symptomatic but content* [33] or *troubled* groups [21,32] displaying the greatest instability. Even if the findings regarding instability are not consistent, those studies provide good insight into patterns of change that occur at certain life stages and thus provide a comprehensive understanding of mental health changes [34].

### Classification approaches for dual-factor mental health

To investigate dual-factor mental health groups, researchers have employed diverse approaches to group individuals based on their dual-factor mental health status, including distinct *cut-score methods* (e.g., [20]) and *latent class analysis* (LCA) or *latent profile analysis* (LPA) (e.g., [32]).

*Cut scores* (for example, sample or norm-based cut-off points) are applied to sort individuals into the four groups of the dual-factor mental health model (psychopathology and PMH above or below the cut score). This can lead to differences between groups that may lack significance or introduce errors in group classification (e.g., [35]).

In comparison, in *latent class analysis* (LCA) or *latent profile analysis* (LPA) – a categorical latent variable mixture modelling approach – individuals are classified based on empirical evidence [36] rather than relying on logically derived criteria [37]. Since LCA/LPA identifies groups based on the data, it does not necessarily result in four groups, as is the case with theory-driven procedures. In several studies using LPA, only three distinct mental health groups were identified [31,38,39]. For example, Jiang et al. [31] and Zhou et al. [38] identified the groups: *complete mental health*, *vulnerable* and *troubled*. Whereas Clark & Malecki [39] identified a different three profile solution corresponding to the groups: *complete mental health*, *symptomatic but content* and *troubled*.

### The present study

Based on the lack of longitudinal dual-factor modelling in the population of medical students, we address the following research questions:

RQ1: Do the identified latent profiles based on the psychopathology and PMH of first-year medical students correspond to the dual-factor model of mental health?

We expected to discover comparable profiles according to the dual-factor model of mental health.

RQ2: How does medical students’ dual-factor mental health change during the first year of medical school?

Our aim was to identify distinct change trajectories, specifically emphasizing the influence of dual-factor mental health status when entering medical school on subsequent changes in mental health during the first year of medical school. This research question was explorative in nature.

## Methods

### Study design

To ensure a larger sample size and increase the generalisability of the results, this study is based on data from two longitudinal online studies with identical content.

- Study I was a multi-centre study in Germany obtaining eight medical schools and took place in 2021/22.
- Study II was repeated one year later (2022/23) at one of the eight medical schools (Centre 1).

Medical students were surveyed with the same questionnaire three times during their first year of medical school: when entering medical school (T0 = Oct 2021 or 2022), at the end of the first semester (T1 = Jan 2022 or 2023) and at the end of the second semester (T2 = Jun 2022 or 2023). Participation was voluntary and incentivised by course credit or monetary compensation. Informed consent was obtained in advance. The methods were carried out according to the Declaration of Helsinki and the European Data Protection Law. This study adhered to ethical standards and was deemed unproblematic by the ethics committee of the medical faculty of the Ludwig-Maximilians-University Munich (approval code: 21–0711).

### Participants

A total of 450 first-year medical students (*n*_T0_ = 299, *n*_T1_ = 287, and *n*_T2_ = 268) from eight medical schools in Germany participated in the online survey. Among the participants, 65.6% were female, 34.0% were male and 0.4% were nonbinary. The mean age of the overall sample was 21.11 years (*SD* = 3.25).

To answer RQ1, we considered all the data available for every measurement point. Only students who participated in the survey three times (*n* = 151) were included in the longitudinal analyses (RQ2). For a detailed description of the investigated subsamples, see Table A1 (supplementary material).

### Measures

The following established scales were used to assess dual-factor mental health:

#### Depression

To capture psychopathology in terms of the dual-factor model of mental health, we measured depression severity using the Patient Health Questionnaire-9 (PHQ-9) [40] (German translation [41]) which consists of nine items. Each item (such as ‘Little interest or pleasure in doing things.’) was rated on a 4-point Likert scale ranging from 0 (*not at all*) to 3 (*nearly every day*), corresponding to one of the nine DSM-IV diagnostic criteria for major depression. The PHQ-9 is widely regarded as a valid and useful instrument for screening depression, both in clinical and non-clinical populations (cf. [42,43]). The reliability assessed by Cronbach’s α in the present study was good at all measurement points (all α ≥ .86; see Table 1 for detailed information).

**Table 1.** Descriptive statistics and correlations of the mental health indicators.

							Pearson correlations								
Variable	*α*	*N*	Min	Max	*M*	*SD*	DE T0	DE T1	DE T2	GLS T0	GLS T1	GLS T2	WB T0	WB T1	WB T2
DE^a^ T0	0.86	297	0.00	2.78	0.67	0.53	–								
DE^a^ T1	0.86	286	0.00	3.00	0.91	0.59	.606**	–							
DE^a^ T2	0.87	268	0.00	2.67	0.92	0.58	.504**	.624**	–						
GLS^b^ T0	–	299	0.00	9.00	6.93	1.63	−.555**	−.418**	−.272**	–					
GLS^b^ T1	–	287	0.00	9.00	6.60	1.77	−.391**	−.528**	−.362**	.566**	–				
GLS^b^ T2	–	268	0.00	9.00	6.51	1.76	−.331**	−.372**	−.577**	.470**	.505**	–			
WB^c^ T0	0.74	299	0.50	4.00	2.69	0.66	−.556**	−.352**	−.413**	.635**	.396**	.356**	–		
WB^c^ T1	0.76	287	0.17	4.00	2.51	0.72	−.462**	−.611**	−.455**	.444**	.676**	.443**	.580**	–	
WB^c^ T2	0.73	268	0.00	4.00	2.45	0.73	−.244**	−.424**	−.632**	.339**	.374**	.657**	.464**	.644**	–

Cronbach’s alpha could not be calculated for GLS because of its single item nature.

*T0* When entering medical school, *T1* End of the first semester, *T2* End of the second semester.

*DE* Depression, *GLS* General Life Satisfaction, *WB* Well-being.

^a^Response range: 0 (not at all) … 3 (nearly every day).

^b^Response range: 0 (entirely unsatisfied) … 10 (entirely satisfied).

^c^Response range: 0 (certainly not) … 4 (yes, exactly like that).

* p < 0.05; ** p < 0.01.

Positive mental health, in the sense of the dual-factor model of mental health, was measured by two indicators: general life satisfaction and well-being.

#### General life satisfaction

General life satisfaction was assessed using the German Single-Item Scale L1 [44]: ‘How satisfied are you at the moment, all in all, with your life?’. This item was rated from 0 (*entirely unsatisfied*) to 10 (*entirely satisfied*). As the formulation of the item closely aligns with its definition, content validity is ensured. Besides, evidence for high construct validity and acceptable test-retest reliability has been provided by Beierlein et al. [44].

#### Well-being

The subscale ‘well-being’ of the FAHW-12 (original instrument in German [45]) was applied to assess medical students’ well-being in accordance to the WHO definition. The six-item subscale assesses psychological (e.g., ‘I am very balanced.’), physical (e.g., ‘I agree with the state of my body.’) and social well-being (e.g., ‘I can approach others without any problems.’) with two items each. The items were answered on a five-point Likert scale from 0 (*certainly not)* to 4 (*yes, exactly like that*). Evidence supporting construct validity of the FAHW-12 has been provided by Wydra [45]. In the present study, the reliability was acceptable at all three measurement points (all α ≥ .73; see Table 1).

### Analysis

We applied IBM SPSS Statistics 29 [46] to conduct descriptive statistics and correlation analyses.

To assess RQ1, exploratory latent profile analyses (LPA) were conducted using M*plus* 8.8 [47] for each measurement point (T0, T1 and T2), testing models with 2-, 3-, 4-, 5- and 6-profile solutions on the data. The continuous indicators included in the LPA were depression severity, general life satisfaction and well-being z-standardized using the mean value at T0. All models were estimated using maximum likelihood robust estimation. To avoid local maxima, random starts for all LPA models were set at 1000, with 500 iterations and 250 optimisation phases [48]. To handle missing data, we utilized maximum likelihood estimation (MLR) with robust standard errors.

The evaluation of model fit was based on the following criteria: Akaike Information Criterion (AIC), Bayesian Information Criterion (BIC), sample-adjusted Bayesian information Criterion (SABIC), Lo-Mendell-Rubin Likelihood Ratio Test (LMR), Bootstrapped Likelihood Ratio Test (BLRT), and entropy. AIC, SABIC and BIC values were used to assess the goodness-of-fit, with smaller values indicating a better fit [36,49]. LMR and the BLRT were used to compare the current number of profiles (k) with a model with k-1 profiles to determine whether the k-profile model was a better fit. A significant test indicates that the k-profile model fits the observed data significantly better than the k-1 profile model [36,49]. Lastly, entropy was used to assess the overall classification quality, with values closer to 1 indicating better model classification [36,49]. When fit indices indicate multiple potential profile solutions, the literature suggests choosing the best solution based on prior theoretical considerations [50].

To address RQ2, we first investigated overall changes in dual-factor mental health indicators by calculating repeated measures ANOVA. We then analysed stability and change patterns descriptively by calculating frequencies (the group assignment was based on the likeliest group membership). Only students who participated three times were included in these analyses.

## Results

### Descriptive statistics and correlations

The average responses for the investigated variables and correlations between the measurement points are presented in Table 1. Depression is strongly negatively related to both PMH indicators at T0, T1 and T2. Strong positive correlations were found between general life satisfaction and well-being at all measurement points.

### Cross-sectional latent profile analysis at all measurement points (RQ1)

**Figure 1.**
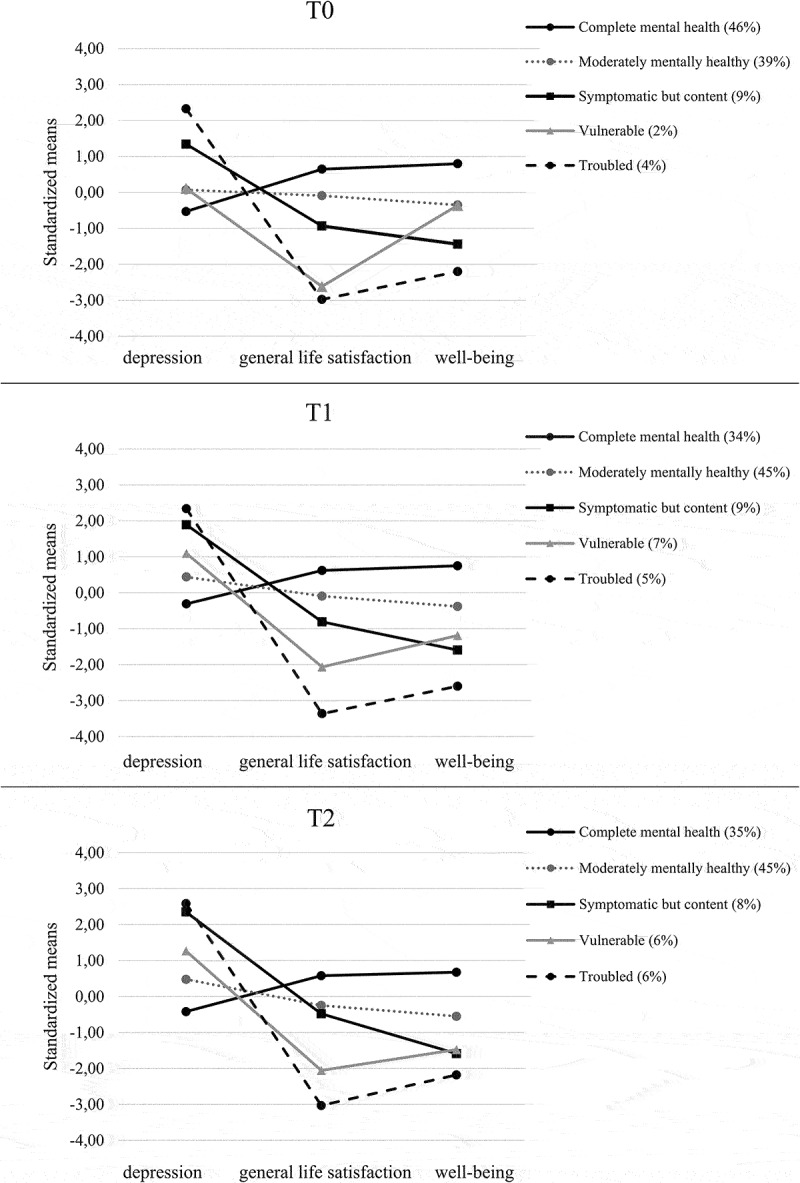
Profile plots displaying means for the five-profile solution.

The fit indices for the 2- to 6-profile solutions are described in Table 2. The fit indices for T1 and T2 were not as clear as for T0, and therefore the 3-, 4-, and 5-profile solutions were further investigated by analysing plots of mean scores for each LPA model at all measurement points (see Figure 1 and Figure A1 supplementary material). The 5-profile model was considered the optimal profile solution because:

1. BIC indices indicated the best fit for the 5-profile solution at T0 and T2 and entropy levels were also satisfactory at these two measurement points.
2. The 5-profile model demonstrated consistent structural similarities at each measurement point. It included all analogous groups as suggested by the dual-factor model, as well as one additional group – the *moderately mentally healthy* group.
3. The classification probabilities for the 5-profile solution (all >0.84 (T0); > 0.77 (T1); >.81 (T2)) also indicate a good fit and high classification certainty.

**Table 2.** Statistics of profile structures.

	No. of Profiles	LL	FP	AIC	BIC	SABIC	LMR (*p*)	BLRT (*p*)	Entropy	Proportion per profile
T0 (*N* = 299)	2	−1130.713	10	2281.427	2318.431	2286.717	**0.0092**	**0.0000**	**0.921**	0.14, 0.86
	3	−1090.806	14	2209.612	2261.418	2217.018	**0.0150**	**0.0000**	0.784	0.08, 0.34, 0.58
	4	−1073.891	18	2183.783	2250.391	2193.306	0.4773	**0.0000**	**0.821**	0.04, 0.05, 0.35, 0.56
	5	−1053.043	22	2150.087	**2231.497**	2161.726	**0.0363**	**0.0000**	**0.812**	0.02, 0.04, 0.09, 0.39.0.46
	6	−1043.464	26	2138.928	2235.139	2152.683	0.7170	**0.0000**	0.800	0.02, 0.04, 0.05, 0.10, 0.35, 0.43
T1 (*N* = 287)	2	−1157.877	10	2335.753	2372.348	2340.637	**0.0003**	**0.0000**	**0.909**	0.18, 0.82
	3	−1115.300	14	2258.600	2309.832	2265.437	**0.0504**	**0.0000**	**0.835**	0.06, 0.25, 0.69
	4	−1093.450	18	2222.901	**2288.772**	2231.692	0.0956	**0.0000**	0.770	0.05, 0.13, 0.40, 0.41
	5	−1084.195	22	2212.391	2292.899	2223.135	0.8473	**0.0000**	0.767	0.05, 0.07, 0.09, 0.34, 0.45
	6	−1074.744	26	2201.488	2296.635	2214.186	0.4760	**0.0000**	0.786	0.02, 0.04, 0.07, 0.09, 0.32, 0.45
T2 (*N* = 268)	2	−1075.608	10	2171.216	2207.126	2175.420	**0.0000**	**0.0000**	**0.900**	0.19, 0.81
	3	−1034.566	14	2097.133	2147.406	2103.018	0.0616	**0.0000**	0.764	0.13, 0.42, 0.45
	4	−1015.136	18	2066.272	2130.910	2073.839	**0.0290**	**0.0000**	0.796	0.07, 0.11, 0.36, 0.45
	5	−1000.880	22	2045.761	**2124.762**	2055.009	0.0902	**0.0000**	**0.829**	0.06, 0.06, 0.07, 0.35, 0.45
	6	−995.494	26	2042.987	2136.353	2053.917	0.4526	0.1132	0.816	0.06, 0.06, 0.06, 0.08, 0.34, 0.40

Note. *LL* log-likelihood. *FP* free parameters. *AIC* Akaike information criterion. *BIC* Bayesian information criterion. *SABIC* sample size – adjusted Bayesian information criterion. *LMR* Lo-Mendell-Rubin likelihood ratio test. *BLRT* bootstrapped likelihood ratio test. Lower AIC, BIC, and SABIC values and higher entropy levels indicate a better profile solution. The LMR and BLRT indices indicate whether the k-profile solution fits the observed data significantly better than the k-1 profile solution. Significant values are highlighted in bold.

Based on the patterns of mean scores across the mental health indicators (Figure 1), we named the 5 groups as follows: (1) *complete mental health* (low depression, high general life satisfaction and high well-being); (2) *moderately mentally healthy* (low depression, high-average general life satisfaction and high-average well-being); (3) *vulnerable* (low-average depression, low-average general life satisfaction and low-average well-being); (4) *symptomatic but content* (high depression, average general life satisfaction and low well-being); and (5) *troubled* (high depression, low general life satisfaction and low well-being).

### Overall changes in mental health indicators

First, we were interested in the overall changes in dual-factor mental health indicators. Repeated measures ANOVA with a Greenhouse-Geisser correction revealed statistically significant differences between measurement points for depression (*F*(1.90, 283.25) = 30.18, *p* < .001, partial η^2^ = .17, *n* = 150), general life satisfaction (*F*(1.84, 275.58) = 10.97, *p* < .001, partial η^2^ = .07, *n* = 151) and well-being (*F*(1.92, 287.29) = 13.08, *p* < .001, partial η^2^ = .08, *n* = 150). Bonferroni-adjusted post-hoc analysis revealed that, at the beginning of medical school (T0), students demonstrated significantly (*p* ≤ .001) lower levels of depression and higher levels of general life satisfaction and well-being, as compared to the end of the first semester (T1) and compared to the end of the second semester (T2) (depression: *M*_DiffT0-T1_ = −0.26, 95%-CI [−0.34, −0.17]; *M*_DiffT0-T2_ = −0.27, 95%-CI[−0.37, −0.16]; general life satisfaction: *M*_DiffT0-T1_ = 0.47, 95%-CI[0.23, 0.72]; *M*_DiffT0-T2_ = 0.50, 95%-CI[0.20, 0.80]; well-being: *M*_DiffT0-T1_ = 0.24, 95%-CI[0.13, 0.35]; *M*_DiffT0-T2_ = 0.20, 95%-CI[0.06, 0.33]).

No differences could be found for the indicators between T1 and T2 (*p* > .05). The means and standard deviations are described in Table 3.

**Table 3.** Means and standard deviations of the dual-factor mental health indicators.

	M	SD
Depression T0	0.60	0.47
Depression T1	0.85	0.56
Depression T2	0.86	0.59
General life satisfaction T0	7.21	1.36
General life satisfaction T1	6.74	1.58
General life satisfaction T2	6.72	1.68
Well-being T0	2.74	0.59
Well-being T1	2.50	0.63
Well-being T2	2.55	0.71

### Descriptive analysis of change trajectories (RQ2)

How mental changes took place, is described in the following.

First, we were interested in the ***stability*** of mental health groups across all three measurement points and the differences between the consecutive measurement points. A total of 38% (*n* = 58) of the sample remained in the same profile over all three measurement points; most of these students were in the *complete mental health* group (60%, *n* = 35) and *moderately mentally healthy* group (38%, *n* = 22). Only one student remained in the *symptomatic but content* group across all measurement points. No stability over all measurement points was found for the *vulnerable* and *troubled* groups.

From T0 to T1, 55% (*n* = 41) of the *complete mental health* group, 63% (*n* = 38) of the *moderately mentally healthy* group, 45% (*n* = 5) of the *symptomatic but content* group, 67% (*n* = 2) of the *vulnerable* group and 50% (*n* = 1) of *the troubled* group remained in the same group. In comparison, between measurement points T1 and T2, 75% (*n* = 40) of the *complete mental health* group, 53% (*n* = 37) of the *moderately mentally healthy* group, 36% (*n* = 5) of the *symptomatic but content*, 18% (*n* = 2) of the *vulnerable* group and 0% of the *troubled* group appeared stable. The percentages need to be interpreted with caution due to the strongly varying group sizes.

**Figure 2.**
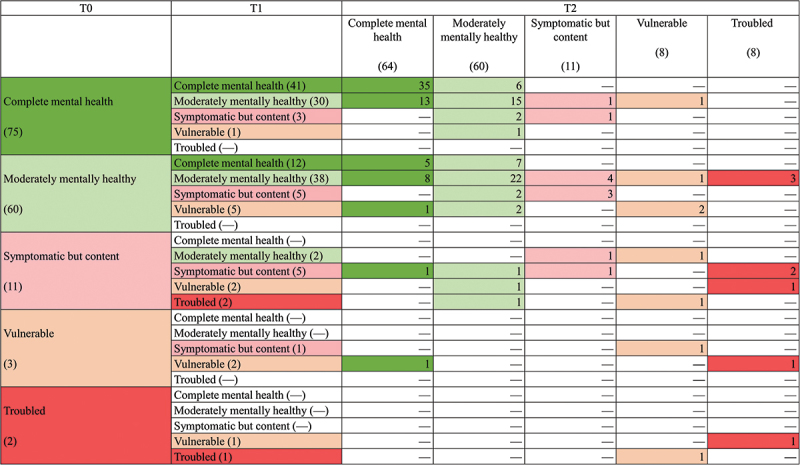
Counts of all change trajectories.

Note. Numbers in brackets show the number of students (*n*),—means that no persons could be assigned to this group; colours for the respective groups were used to highlight the change trajectories. The sub-groups were colour-coded to make the trajectories of change easier to identify (green shades = mentally healthy groups; red shades = groups at risk).

Second, we were interested in ***change trajectories***. Overall, various change trajectories can be observed, pointing towards both recovery (for example, from *symptomatic but content* to *moderately mentally healthy*) and deterioration (for example, from *complete mental health* to *vulnerable*). All change trajectories and their counts can be found in Figure 2.

The analysis of change trajectories revealed that students who showed *complete mental health* at the beginning of medical school did not change into the *troubled* group at any time. In addition, if a student is in the *complete mental health* group at the end of the first semester (T1), the apparent probability is high that one will remain in this group (T2, 75%) or only move into the *moderately mentally healthy* group, but no changes into other groups could be observed from T1 to T2. In general, many students who enter medical school with *complete mental health* (T0) are either in the *moderately mentally healthy* or *complete mental health* group at the end of the second semester (T2). Only a few exceptions show mental health patterns at T2 that correspond to the *vulnerable* or *symptomatic but content* group (only 3 out of 75) after one year at medical school.

Furthermore, of the 30 students who slipped from the *complete mental health* group to the *moderately mentally healthy* group (T0 → T1), almost half (*n* = 13) showed a recovery pattern (T1 → T2). The rest (*n* = 15) remained in the *moderately mentally healthy* group and only a very small part deteriorated into the *vulnerable* and *symptomatic but content group* (one person each).

Changes from the groups *symptomatic but content*, *vulnerable* and *troubled* to the *complete mental health* group could not be demonstrated between T0 and T1; however, changes from the groups *symptomatic but content* and *vulnerable* to the *complete mental health* group could be demonstrated between T1 and T2. The interpretation of these results should be taken with caution, as they are only individual cases (*n* = 3).

## Discussion

The dual-factor model of mental health highlights the significance of assessing psychopathology and positive mental health (PMH) concurrently to offer a more comprehensive understanding of mental health [28]. However, there is limited knowledge about dual-factor mental health in medical students and its changes during the first year of medical school – a critical period for medical students’ mental health [1–4].

This study primarily seeks to explore the development of dual-factor mental health from entering medical school to the end of the first academic year to better understand the impact of medical training on mental health changes.

### Dual-factor mental health groups

Applying latent profile analysis, we identified five mental health groups across the three measurement points. In addition to the four groups that corresponded to the dual-factor model of mental health, (1) *complete mental health*, (2) *symptomatic but content*, (3) *vulnerable*, (4) *troubled*, we identified an additional group – the (5) *moderately mentally healthy* group. The *moderately mentally healthy* group is characterized by high PMH and low psychopathology but less pronounced compared to the *complete mental health* group. Moore et al. [32,51] also identified a *moderately mentally healthy* group among adolescents. The difference was that they identified a 4-profile solution including *complete mental health*, *moderately mentally healthy*, *symptomatic but content* and *troubled* groups but did not find support for the *vulnerable* group. Yet another study investigating dual-factor mental health among children also identified a 5-profile solution, but these resulted in partly different groups in terms of content: *complete mental health, conduct problems but content, emotional symptoms but content*, *vulnerable* and *troubled* [34]. Again, in other investigations, only three profiles were identified. For instance, two studies could not detect the *symptomatic but content* group in their sample [31,38], while another study could not detect the *vulnerable* group [39]. The different profile solutions may be due to the different investigated populations and the different mental health indicators used in previous studies. While our study outcomes may deviate from the four sub-groups outlined in the dual-factor model, they nonetheless lend support to the framework of the dual-factor model, as all four groups of the model were identified.

### Dual-factor mental health changes during the first year

In total, 62% of the students showed changes in their mental health status during the first year of medical school, which means that only 38% remained in their initial mental health group across all measurement points. In line with studies in other populations [19–21,33,51], the *complete mental health* group emerged as the most common (50% at T0) and the most stable group (47% remained in the same group at all measurement points). This means that half of the investigated sample started medical school with *complete mental health*. Of these, around half remained in this group across all measurement points. This suggests that the *complete mental health* group copes better with academic challenges during their first year of medical school.

Comparing the stability patterns of *complete mental health* between the measurement points, it seems that for students who are still in the *complete mental health* group at T1, the probability appears high that they will remain in this group (75%) – i.e., for students who got through the first semester well, the probability is high that they will continue to do so. Furthermore, students who showed *complete mental health* at the beginning of medical school did not change into the *troubled* group at any time. Thus, initial *complete mental health* seems to be a protective factor for the development of later mental health difficulties during the initial year of medical school. Moreover, students with initial *complete mental health* have a high tendency to recover when they show changes between the first two measurement points, which may be due to differences in individual characteristics when entering medical school. This is in line with the authors’ previous research, revealing that students who did not show any signs of depression during the first semester had higher self-efficacy and resilience when entering medical school compared to those who experienced an increase in depressive symptoms [1]. Another reason for the high stability and tendency to recover in the *complete mental health* group may be due to their potentially higher levels of grit [29] and locus of control [25,29]. These are important factors in coping with a high workload, as it is the case at medical school.

Compared to the *vulnerable* and *troubled* groups, the *symptomatic but content* group showed a higher probability of recovering – possibly due to higher levels of general life satisfaction. However, this interpretation should again be treated with caution because of the small group size.

The *vulnerable* and *troubled* groups proved to be the least stable between all measurement points. This is in accordance with previous studies that have reported low stability for the *vulnerable* [19,20,34] and the *troubled* [21] groups. Moreover, previous studies indicate that individuals in the *vulnerable* group are more likely to encounter academic difficulties [22] and experience less joy and more frustration while learning compared to their counterparts in the *complete mental health* group [21]. Besides, the troubled group was previously found to be associated with lower self-efficacy [21]. These are possible reasons for the lower stability.

In addition to discussed changes that indicate a deterioration in mental health, there were individual cases that showed an improvement in mental health during the first year (for example, *vulnerabl*e at T0 and *complete mental health* at T2). The reasons for the improvements could be the elimination of additional burdens or individual crises that existed when entering medical school. Alternatively, the initial phase may have been perceived as stressful due to the numerous demands of the new environment, but a favorable adjustment occurred promptly.

### Implications for research and practice

We derive the following implications for research and practice from our findings:

1. Our results have shown that initial mental health status is an important factor in predicting future mental health changes. Therefore, to better understand changes in mental health during medical school, it is important to consider students’ initial mental health status.
2. Preventive measures should be integrated into the curriculum as early as the first year of medical school to prevent deteriorations in mental health and their potential impact on learning and professionalism.
3. When designing preventive measures, it is important to consider that some students enter medical school with impaired mental health. Low-threshold psychiatric-psychotherapeutic counselling services should be established for those students.
4. Particular attention should be paid to the *moderately mentally healthy*, *vulnerable*, *symptomatic but content* and *troubled* groups, as these do not have a high recovery potential like the *complete mental health* group. One preventive strategy could be (near-)peer mentoring programmes. Qualitative studies have shown that peer mentoring programmes in the first year are perceived as helpful in adapting to campus life (cf. [52]). The results suggest that such programmes can reduce stress and enhance resilience [53,54].
5. Future research should investigate which factors (especially factors associated with medical education) predict dual-factor mental health group membership and changes that occur during medical school. These results can serve as the basis for tailored preventive measures.

### Strengths and limitations

This study provides valuable insights into the heterogeneity of medical students’ initial dual-factor mental health status and changes during the first year of medical school. Nevertheless, several factors limit the generalizability of our results. Our study was based on a moderate sample of students from eight German medical schools. The sample sizes of some mental health groups were very small (for example, *troubled*), limiting the interpretability of the stability and change patterns for these groups. The dropout rate could not be determined due to the different composition of participants per measurement point, but it can be assumed that the actual number of students in the groups – *symptomatic but content*, *vulnerable* and *troubled* is higher than proven in our study. Our results should be replicated with a larger sample. Furthermore, mental health underlies multiple influences (e.g., [55]); therefore, influences external to medical school cannot be excluded.

## Conclusion

Our findings improve the understanding of mental health changes in the first year of medical school by revealing distinct mental health trajectories. Our results indicate that mental health changes are largely dependent on initial mental health status when entering medical school. Students in the *complete mental health* group had a higher potential to recover after deteriorations in the first semester compared to their peers. To summarize, our results highlight the tremendous heterogeneity in mental health changes in medical students that militate against a one-size-fits-all prevention strategy.

## Supplementary Material

Supplemental Material

## Data Availability

The data that support the findings of this study are available from the corresponding author, [SP] upon reasonable request. The data are not publicly available due to privacy and data storage restrictions on university internal servers.
