# Supplementary material for: Uncovering heterogeneity in mental health changes among first-year medical students
Source: Med Educ Online. 2024 Feb 23;29(1):2317493. doi: 10.1080/10872981.2024.2317493 (PMC10896144; doi:10.1080/10872981.2024.2317493)
Supplement: Supplemental Material [file ZMEO_A_2317493_SM8092.zip › Supplementary files/FigureA1_Caption_Supplemental online material.docx]

## Supplemental online material

**Figure A1**. Plots of mean scores for the 3-and 4-profile LPA models at all measurement points
