# Supplementary material for: Uncovering heterogeneity in mental health changes among first-year medical students
Source: Med Educ Online. 2024 Feb 23;29(1):2317493. doi: 10.1080/10872981.2024.2317493 (PMC10896144; doi:10.1080/10872981.2024.2317493)
Supplement: Supplemental Material [file ZMEO_A_2317493_SM8092.zip › Supplementary files/TableA1_Supplemental online material_revised.docx]

## Supplemental online material

# Table A1. Sociodemographic information of the investigated subsamples reported as mean ± standard deviation or *n* (%)

| Variable | Overall  *N* = 450 | T0 (RQ1)  *n* = 299 | T1 (RQ1)  *n* = 287 | T2 (RQ1)  *n* = 268 | T0T1T2 (RQ2)  *n* = 151 |
| --- | --- | --- | --- | --- | --- |
| *Gender*  female  male  nonbinary | 295 (65.6)  153 (34.0)  2 (0.4) | 200 (66.9)  97 (32.4)  2 (0.7) | 182 (63.4)  104 (36.2)  1 (0.3) | 172 (64.2)  95 (35.4)  1 (0.4) | 96 (63.6)  54 (35.8)  1 (0.7) |
| *Age* (*n* = 449)  Range | 21.11 **±** 3.25  16 – 43 years | 20.97 **±** 2.80  16 – 37 years | 20.90 **±** 3.13  16 – 43 years | 21.09 **±** 3.27  16 – 43 years (*n* = 267) | 20.75 **±** 2.78  16 – 37 years |
| *Medical school (centre)*  1  2  3  4  5  6  7  8 | 190 (42.2)  105 (23.3)  6 (1.3)  18 (4.0)  49 (10.9)  45 (10.0)  21 (4.7)  16 (3.6) | 161 (53.8)  56 (18.7)  4 (1.3)  6 (2.0)  20 (6.7)  27 (9.0)  12 (4.0)  13 (4.3) | 159 (55.4)  52 (18.1)  1 (0.3)  7 (2.4)  31 (10.8)  21 (7.3)  10 (3.5)  6 (2.1) | 162 (60.4)  33 (12.3)  3 (1.1)  7 (2.6)  12 (4.5)  37 (13.8)  7 (2.6)  7 (2.6) | 127 (84.1)  5 (3.3)  1 (0.7)  --  1 (0.7)  12 (7.9)  3 (2.0)  2 (1.3) |
| *University entrance qualification grade*  *range*^1^ | 1.59 **±** 0.55  1.0 – 3.70 | 1.57 **±** 0.53  1.0 – 3.50 | 1.58 ± 0.54  1.0 – 3.5 | 1.64 ± 0.56  1.0 – 3.5 | 1.62 ± 0.51  1.0 – 3.30 |

^1^In the German grading system, A-level grades range from 1.0 to 4.0 (1.0 = very good, 2.0 = good, 3.0 = satisfactory, 4.0 = sufficient).
